# Profiles of job satisfaction among industrial workers and its association with mental health under the background of Industry 5.0 transformation: a latent profile analysis

**DOI:** 10.3389/fpubh.2026.1772767

**Published:** 2026-03-06

**Authors:** Shang Gao, Qiyuan Wang, Keyao Kang, Yuxin Chen, Xiangchen Kong, Xihe Yu

**Affiliations:** School of Public Health, Jilin University, Changchun, Jilin, China

**Keywords:** anxiety, depression, digital-intelligence job insecurity, industrial workers, job satisfaction

## Abstract

**Background:**

Job satisfaction is a critical factor influencing workplace efficiency and employee well-being. In the context of Industry 5.0 transformation, understanding the latent profiles of job satisfaction and their relationship with mental health outcomes, such as depression, anxiety, and digital-intelligence job insecurity, is critical for promoting employee well-being and organizational sustainability. This study aims to explore the latent profiles of job satisfaction among industrial workers and explore their associations with mental health outcomes.

**Methods:**

This study used cross-sectional data from 3,420 male frontline workers from a large automobile manufacturing enterprise in Jilin Province, China in April 2024. Latent profile analysis (LPA) was employed to identify distinct latent profiles of job satisfaction among industrial workers, while hierarchical linear regression analysis was used to analyze the relationship between job satisfaction and psychological health outcomes (depression, anxiety and digital-intelligence job insecurity).

**Results:**

The score of job satisfaction among industrial workers in Jilin Province was 3.62 ± 0.90. Four profiles were identified: very low (5.97%), low-to-moderate (31.14%), moderately high (42.63%), and high job satisfaction (20.26%). Depression and anxiety showed a clear level-gradient pattern across profiles, whereas digital-intelligence job insecurity displayed a non-monotonic pattern with higher levels in the low-to-moderate and moderately high profiles. Work stress showed consistent associations with all outcomes, and job satisfaction profiles remained associated with depression and anxiety after covariate and stress adjustment; associations with digital-intelligence job insecurity were smaller but detectable.

**Conclusion:**

This study examined heterogeneity in job satisfaction among frontline industrial workers and its associations with mental health outcomes. Latent profile analysis identified four job satisfaction profiles. Job satisfaction profile membership remained strongly associated with depression and anxiety. Digital-intelligence job insecurity showed a non-monotonic pattern across profiles. These findings suggest that an individual-centered profile approach provides actionable differentiation of mental health symptom burden across distinct job satisfaction patterns, supporting more targeted workplace strategies.

## Introduction

Job satisfaction is considered the core of successful human resource management (1) and has always been a hot topic in academia and industry (2). High job satisfaction is associated with increased employee productivity, enhanced loyalty, reduced turnover, and improved organizational performance and competitiveness (3–7). Industrial workers, who constitute approximately 24% of the global workforce (8), are a critical driver of economic growth. Therefore, their job satisfaction is expected to remain at a relatively ideal level. However, their job satisfaction often remains suboptimal due to long working hours, monotonous tasks, stringent performance evaluations, occupational hazards, and other workplace challenges (4, 9–13). For instance, over 90% of employees in the automotive manufacturing sector reportedly exhibit only moderate levels of job satisfaction (14). This has serious implications, including poor physical and mental health of employees (15, 16), high turnover rate (17), decreased productivity performance (18, 19), etc.

Mental health issues, particularly depression and anxiety, are increasingly prominent challenges worldwide, with industrial workers identified as a high-risk group (20–23). Studies indicate that the prevalence of depression among industrial workers can reach 21%, significantly exceeding that of the general population (24). Workplace anxiety has also surged globally (25). In addition, when discussing the mental health of industrial workers, the current context of the industry’s transition from Industry 4.0 to Industry 5.0 cannot be ignored (26), these challenges are further compounded by the integration of advanced technologies such as artificial intelligence (AI), robotics, and the Internet of Things (IoT) (26). While such innovations promise new job opportunities, they also heighten concerns about job insecurity. A global survey by the International Federation of Robotics revealed that 42% of respondents expressed anxiety about job loss due to automation (27). Job insecurity, particularly in the context of AI-driven automation, has emerged as a pressing occupational psychological issue (28, 29).

Job satisfaction, as an overarching construct reflecting an individual’s attitude toward work (30), is closely linked to mental health (31). A wealth of research has established that job satisfaction is significantly associated with workers’ psychological well-being, including depression and anxiety (32). Low job satisfaction is associated with higher risks of mental health problems, whereas positive mental health can reinforce job satisfaction (33). Furthermore, job satisfaction plays a critical role in mitigating job insecurity and its adverse outcomes (34–36).

To clearly explain job satisfaction and worker mental health issues, including job insecurity, this study uses the Job Demand-Resource (JD-R) model as its theoretical framework (37). The JD-R model posits that job demands (such as high-intensity labor and skill transitions) deplete individual resources and induce stress responses, which, over time, further damage physical and mental health (38). Conversely, the job resources an individual possesses can buffer work stress and promote work motivation (39, 40). From the JD-R perspective, job satisfaction can be understood as an employee’s subjective evaluation of their total available job resources (41, 42). When employees believe their job resources are insufficient to meet job demands, satisfaction declines (43), and individuals are more likely to experience health deterioration, manifesting as anxiety, depression, and other psychological distress (44). Within the JD–R framework, work stress can be conceptualized as a strain response that lies between job demands and outcome variables (45, 46). Job demands trigger stress-related strain and, in turn, impair mental health. Accordingly, work stress is theoretically linked to both job satisfaction and mental health outcomes, a notion that has also been supported by substantial empirical evidence (47–50). Moreover, because workers may face different configurations of job demands and job resources, the JD–R framework implies that job satisfaction may not be uniform but may cluster into qualitatively distinct patterns across individuals.

Despite these understandings, some research gaps remain. First, most existing research neglects industrial workers, especially frontline employees in manufacturing. Secondly, most studies employ a variable-centric approach, assuming that all workers’ experiences are homogeneous, which ignores the heterogeneity of different individuals in their combinations of job satisfaction dimensions. This heterogeneity may be even more pronounced in the automotive manufacturing industry. This is because job responsibilities in the automotive industry are particularly well-defined, and the work content and environments in different processes (such as welding, painting, final assembly, and equipment maintenance) are entirely different, resulting in significant differences in job performance. Therefore, under the same transformation pressures, workers may also develop different “needs-resource allocations,” manifesting as differentiated combinations of satisfaction items, rather than simply high or low levels.

Accordingly, consistent with the Job Demands–Resources (JD–R) framework, we hypothesize that: (H1) Job satisfaction can be represented by multiple distinct latent profiles; (H2) Depression, anxiety, and digital-intelligence job insecurity differ significantly across job satisfaction profiles. In addition, given the potential confounding role of work stress in the JD–R process, we further test whether the associations between job satisfaction profiles and mental health indicators remain robust after adjusting for key demographic characteristics and work stress.

Taken together, this study aims to identify distinct job satisfaction profiles among frontline automotive manufacturing workers and to examine whether these profiles differ in depression, anxiety, and digital-intelligence job insecurity, while accounting for key demographic characteristics and work stress. These findings may inform targeted workplace policies and interventions to reduce mental health risks during digital-intelligent transformation.

## Methods

### Study design and participants

Based on the core focus of this study: identifying latent profiles of job satisfaction among frontline workers under Industry 5.0 transformation and examining how these profiles relate to mental health outcomes (anxiety, depression, and digital-intelligence job insecurity), the research context needed to meet two conditions: (a) the impact of digital and intelligent transformation on frontline work must be sufficiently significant to trigger and capture digital job insecurity; and (b) job roles and production processes should be sufficiently differentiated to generate heterogeneity in job satisfaction that can be detected through latent profile analysis.

The automotive manufacturing industry satisfies both conditions. It features long supply chains, rapid technological iteration, and intensive digitalization and intelligentization, making it a prototypical setting in which Industry 5.0 transformation pressures are strongly manifested. Meanwhile, its clearly differentiated roles and workflows (e.g., welding, painting, final assembly, and equipment maintenance) provide a natural basis for the emergence of distinct combinations of satisfaction characteristics, which is well suited to latent profile analysis. In addition, the workforce in China’s automotive manufacturing industry is sizable (approximately 4.946 million employees as of November 2025), and identifying job satisfaction profiles in this population has substantial practical relevance and potential for broader implications.

Given sample accessibility, we ultimately selected a large multinational automobile manufacturing enterprise in Jilin Province. This enterprise has widely deployed digital-intelligent equipment and automated production lines across multiple production stages and has introduced a small number of humanoid robots. As a result, frontline workers interact with digital-intelligent systems more frequently in their daily work, making concerns about being replaced by such technologies more salient, thereby facilitating the measurement and testing of digital-intelligence job insecurity and its associations with mental health outcomes.

This study used a cross-sectional design. It was approved by the Ethics Committee the School of Public Health, Jilin University (IRB code 20240409) and was conducted in April 2024 at this automobile manufacturing enterprise in Jilin Province, China. Online questionnaires were distributed to employees through the company’s human resources department. The purpose and objectives of the survey were explained in detail to all participants, and informed consent was obtained prior to participation. Stratified random cluster sampling was employed to ensure sample diversity and representativeness, with participants drawn from 3 plant areas and 12 workshops. A total of 4,000 questionnaires were distributed, and after excluding responses with excessively short completion times or inconsistent logic, 3,420 valid questionnaires were included in the analysis, yielding an effective response rate of 85.5%.

### Instruments and measurements

#### Job satisfaction

Job satisfaction was assessed using the Job Satisfaction Inventory Scale developed by Hackman and Lawler (51). The scale comprises 13 items scored on a 5-point Likert scale (1 = strongly disagree; 5 = strongly agree). The Cronbach’s alpha for this scale in this study was 0.967, indicating excellent reliability.

#### Depression

Depression was measured using the PHQ-9 scale (52), which includes nine items assessing the frequency of depressive symptoms over the past 2 weeks on a 0–3 Likert scale (0 = not at all; 3 = nearly every day). The total score ranges from 0 to 27. In this study, the PHQ-9 demonstrated strong internal consistency, with a Cronbach’s alpha of 0.925.

#### Anxiety

Anxiety was measured using the GAD-7 scale (53), which consists of seven items assessing the frequency of anxiety symptoms over the past 2 weeks on a 0–3 Likert scale. The total score ranges from 0 to 21. The Cronbach’s alpha for the GAD-7 in this study was 0.947, indicating high reliability.

#### Digital-intelligence job insecurity

Digital-Intelligence Job Insecurity Scale used in this study was self-developed based on the foundational work of Ashford et al. (54) and Hellgren et al. (55). This scale was designed to assess job insecurity specifically related to the application of artificial intelligence and intelligent automation technologies. It encompasses three dimensions: (1) job replacement insecurity, (2) job transformation insecurity, and (3) job interaction insecurity, with each dimension comprising three items. Responses were scored on a 5-point Likert scale (1 = strongly disagree; 5 = strongly agree). The overall Cronbach’s alpha for the scale was 0.941, with subscale reliabilities of 0.867, 0.934, and 0.881, respectively, demonstrating good internal consistency. Confirmatory factor analysis supported the proposed structure. We conducted a confirmatory factor analysis using a second-order factor model. The model showed acceptable fit [*χ*^2^(21) = 344.349, CFI = 0.988, TLI = 0.980, RMSEA = 0.067, SRMR = 0.018], and all standardized loadings were significant (0.689–0.948), with significant second-order loadings (0.850–0.938). All CFA results (standardized factor loadings and error variances), the inter-factor correlation matrix, the HTMT matrix, and the CR/AVE calculations are reported in Appendix Tables S1–S3.

#### Work stress

Work stress was assessed using a 13-item challenge–hindrance work stressor measure originally developed by Cavanaugh et al. (56). Items were rated on a 5-point scale ranging from 1 (no stress) to 5 (a great deal of stress) (56, 57). Item scores were averaged to create an overall work stress score, with higher scores indicating greater perceived work stress. The scale demonstrated excellent internal consistency in the present sample (Cronbach’s *α* = 0.963).

### Statistical analysis

Descriptive analysis was conducted to summarize demographic variables. Frequency was used to describe categorical variables.

Latent profile analysis (LPA) was conducted to identify distinct subtypes of job satisfaction among workers. We estimated models with 2–5 profiles and evaluated model fit using the Akaike Information Criterion (AIC), Bayesian Information Criterion (BIC), and sample-size adjusted BIC (aBIC), where smaller values indicate better relative fit. Classification quality was assessed using entropy, with values closer to 1 indicating clearer separation between profiles.

We examined predictors of job satisfaction profile membership using multinomial logistic regression, treating the latent profiles derived from the LPA as a nominal categorical outcome. Profile 1 was specified as the reference category. To determine whether adding an additional profile significantly improved model fit, we used the Lo–Mendell–Rubin adjusted likelihood ratio test (adjusted LMR-LRT), which compares the k-profile model against the (*k* − 1)-profile model; a significant *p*-value suggests the k-profile solution fits better.

A set of demographic and job-related variables were entered simultaneously as predictors, including age group, marital status, fertility (having children), education level, managerial position, technical level, years of employment, monthly income category, average daily working hours, number of night shifts, and work stress. For each non-reference profile, the model estimated odds ratios (ORs) and 95% confidence intervals (CIs) comparing the odds of membership in that profile versus Profile 1. Statistical significance was evaluated using two-sided tests with an alpha level of 0.05. Categorical predictors were dummy-coded using the lowest/most common category as the reference (e.g., 0–3,000 yuan for monthly income; >8 h/day for working hours; no night shifts for night-shift frequency; junior for technical level).

We examined whether depression, anxiety, and digital-intelligence job insecurity differed across demographic and job-characteristic groups. For binary grouping variables (e.g., marital status, fertility status, education level, managerial position), we conducted independent-samples t tests. For variables with three or more categories (e.g., age group, technical level, years of employment, monthly income, average daily working hours, and night-shift frequency), we conducted one-way analyses of variance (ANOVA). Assumptions were evaluated prior to inference. Homogeneity of variances was tested using Levene’s test. When the equal-variance assumption was violated, we reported Welch-corrected *t*-tests for two-group comparisons and Welch’s ANOVA for multi-group comparisons. All tests were two-tailed, with statistical significance set at *p* < 0.05. Descriptive statistics are presented as mean ± standard deviation (SD).

To test whether mental health outcomes varied by job satisfaction profile, we treated latent profile membership as the grouping variable and conducted one-way ANOVA with depression, anxiety, and digital-intelligence job insecurity as dependent variables. Because variance homogeneity was not met, we used Welch’s ANOVA for the omnibus tests. Where omnibus differences were significant, we conducted Games–Howell *post hoc* comparisons, which are robust under unequal variances and unequal sample sizes. Results are reported as Welch’s *F* statistics with corresponding *p* values, and *post hoc* pairwise contrasts are summarized using letter superscripts to indicate statistically distinguishable groups (*p* < 0.05).

We used hierarchical linear regression to examine the incremental contribution of work stress and job satisfaction profiles in predicting depression, anxiety, and digital-intelligence job insecurity. For each outcome, predictors were entered in three steps. In Step 1, we entered demographic and job-characteristic covariates. In Step 2, we added work stress. In Step 3, we added job satisfaction profile membership as a set of dummy variables, using Profile 1 as the reference group. We evaluated incremental predictive utility using the change in explained variance (Δ*R*^2^) at each step and the corresponding F-change tests. Regression coefficients are reported as unstandardized B with 95% confidence intervals (CIs) and *p*-values. All tests were two-tailed with *p* < 0.05 indicating statistical significance.

Analyses were conducted using IBM SPSS Statistics 26 and Mplus 8.0.

## Results

### Sociodemographic characteristics of the respondents

Table 1 summarizes the sociodemographic characteristics of the study participants. All respondents in this study were male. The majority (60.1%) were aged between 18 and 30 years, followed by 26.9% aged 31–45 years and 13.0% aged 46–60 years. Most respondents were unmarried (60.7%), while 39.3% were married. Regarding fertility status, 67.1% of the workers had no children, and 32.9% had children. In terms of education, 86.7% of the respondents had a college degree or above, while 13.3% had a high school education or below. A small proportion of workers (9.6%) held managerial positions, whereas the majority (90.4%) did not. Regarding technical qualifications, 60.1% of workers were classified as junior level, 29.2% as intermediate, and 10.7% as senior. The length of employment varied, with 70.6% of workers having worked for 1–10 years, 16.7% for 10–20 years, and 12.6% for over 20 years. Monthly income distribution showed that 16.2% of respondents earned 0–3,000 yuan, 56.0% earned 3,000–5,000 yuan, 23.2% earned 5,000–8,000 yuan, and 4.6% earned more than 8,000 yuan.

**Table 1:** Sociodemographic characteristics of the respondents.

Characteristics	*n* (%)
Age	
18–30	2057 (60.1)
31–45	920 (26.9)
46–60	443 (13.0)
Marital status	
Unmarried	2075 (60.7)
Married	1,345 (39.3)
Fertility status	
No children	2,296 (67.1)
Have children	1,124 (32.9)
Education	
High school and below	456 (13.3)
College degree and above	2,964 (86.7)
Management position	
Yes	328 (9.6)
No	3,092 (90.4)
Technical level	
Junior	2056 (60.1)
Intermediate	999 (29.2)
Advanced	365 (10.7)
Work years	
0–10	2,416 (70.6)
10–20	572 (16.7)
>20	432 (12.6)
Monthly income (yuan)	
0–3,000	555 (16.2)
3,000–5,000	1916 (56.0)
5,000–8,000	793 (23.2)
>8,000	156 (4.6)
Average daily working hours (h)	
<8	45 (1.3)
8	468 (13.7)
>8	2,907 (85.0)
Number of night shifts (times)	
0	1,080 (31.6)
1	97 (2.8)
2	774 (22.6)
≥3	1,469 (43.0)

### Fit statistics for latent profile analysis

In the latent profile analysis, we compared solutions with two to five profiles (fit indices are shown in Table 2; class counts and proportions are reported in Appendix Table S4). As the number of profiles increased, AIC, BIC, and sample-size adjusted BIC (aBIC) decreased monotonically, and classification quality remained high across solutions (entropy = 0.958–0.972). The four-profile solution showed strong classification quality (entropy = 0.972), with class proportions of 5.97% (*n* = 204), 31.14% (*n* = 1,065), 42.63% (*n* = 1,458), and 20.26% (*n* = 693); the smallest class accounted for 5.97%, which is generally acceptable. In contrast, although the five-profile solution further reduced information criteria, it produced a notably small class (3.15%, *n* = 108), suggesting potential over-extraction or an unstable class. Moreover, the adjusted Lo–Mendell–Rubin likelihood ratio test (LMR-LRT) indicated that the five-profile solution did not fit significantly better than the four-profile solution (*p* = 0.087). From a theoretical perspective, the four-profile solution displayed a clear overall “severity-gradient” pattern: the profile curves were similar in shape with minimal crossover, and differences were primarily reflected in overall satisfaction levels rather than structural reversals across dimensions. Accordingly, we labeled the profiles as “very low,” “low-to-moderate,” “moderately high,” and “high” job satisfaction. Taken together, we retained the four-profile solution as the final model.

**Table 2:** Fit statistics for latent profile analysis.

Models	AIC	BIC	aBIC	Entropy	Adjusted LMR-LRT	*p*
2	107588.087	107833.583	107706.484	0.958	23416.709	<0.001
3	98516.375	98847.794	98676.211	0.965	9020.531	<0.001
4	88708.071	89125.414	88909.346	0.972	9750.714	<0.001
5	86623.452	87126.718	86866.166	0.970	2094.237	0.087

AIC = Akaike information criterion; BIC = Bayesian information criterion; aBIC = sample-size adjusted BIC; Adjusted LMR-LRT = Lo–Mendell–Rubin adjusted likelihood ratio test.

**Figure 1:**
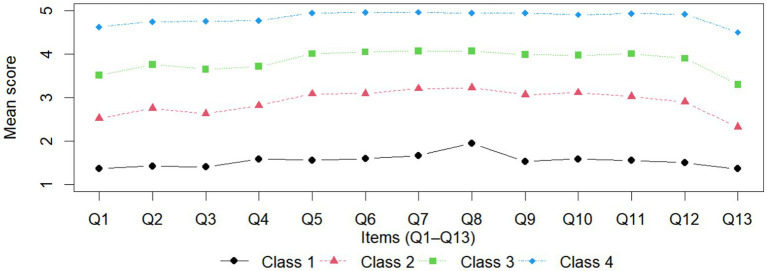
Latent profiles of job satisfaction. Profile 1 = very low job satisfaction group, Profile 2 = low-to-moderate job satisfaction group, Profile 3 = moderately high job satisfaction group, and Profile 4 = high job satisfaction group.

The overall mean job satisfaction score of the sample population was 3.62 (SD = 0.900). Figure 1 displays the mean scores for each item across the four latent profiles of job satisfaction, highlighting their distinct characteristics.

We named the four profiles very low, low-to-moderate, moderately high, and high job satisfaction group. This naming is based on the fact that the four categories show a consistent, unidirectional change in the mean values of items Q1–Q13, and the profile curves are similar in shape, almost parallel, and with minimal crossover across items, suggesting that the category differences primarily reflect “severity” rather than “structure.” Furthermore, the mean values for each category show a clear gradient distribution: Profile 1’s mean is concentrated at the low end of the scale (approximately 1.36–1.95), Profile 2 is in the low to medium range (approximately 2.32–3.22), Profile 3 reaches the medium-high range (approximately 3.29–4.07), and Profile 4 is close to the high end of the scale (approximately 4.50–4.97). Therefore, using overall-level naming not only better reflects the underlying structure reflected by the data but also avoids over-interpreting certain specific dimensions, thereby improving the transparency, interpretability, and practical usability of the results.

#### Profile 1: very low job satisfaction group

Profile 1 (the Very Low Job Satisfaction Group) showed the lowest overall job satisfaction among the four profiles, with a mean score of 1.54 (SD = 0.45). At the item level, this profile had the lowest mean on Q13 (1.36 ± 0.62) and the highest mean on Q8 (1.95 ± 1.12). This group included 204 workers, accounting for 5.97% of the sample.

#### Profile 2: low-to-moderate job satisfaction group

Profile 2 (the Low-to-moderate Job Satisfaction Group) showed the relatively low overall job satisfaction among the four profiles, with a mean score of 2.90 (SD = 0.27). At the item level, this profile had the lowest mean on Q13 (2.32 ± 0.89) and the highest mean on Q8 (3.22 ± 0.71). This group included 1,065 workers, accounting for 31.14% of the sample.

#### Profile 3: moderately high job satisfaction group

Profile 3 (the Moderately High Job Satisfaction Group) exhibited relatively high overall job satisfaction, with a mean score of 3.84 (SD = 0.25). Item scores were consistently elevated, ranging from 3.29 to 4.07, indicating broadly high satisfaction across domains. Profile 3 included 1,458 workers, representing 42.63% of the sample.

#### Profile 4: high job satisfaction group

Profile 4 (the High Job Satisfaction Group) demonstrated the highest overall job satisfaction, with a mean score of 4.84 (SD = 0.22). Scores across all items remained very high (range: 4.62–4.96), suggesting a stable and comprehensive pattern of high satisfaction. This profile included 693 workers, accounting for 20.26% of the sample.

### Latent profile differences by characteristics

Table 3 presents the distribution of demographic characteristics across the four latent profiles. Significant between-profile differences were observed in age, marital status, fertility status, education, management position, technical level, years of work, and monthly income (all *p* < 0.05; most *p* < 0.001). Across all profiles, workers aged 18–30 years constituted the largest proportion, and the majority were unmarried, without children, at a junior technical level, and with 0–10 years of work experience. Regarding education, all profiles were predominantly composed of workers with a college degree and above (85.0–89.3%). Notably, Profile 3 had the highest proportion of workers with a high school education or below (15.0%), whereas Profile 4 had the lowest (10.7%). For management position, non-management workers accounted for the overwhelming majority in all profiles (87.8–97.1%), with Profile 1 showing the lowest proportion of management positions (2.9%) and Profile 3 the highest (12.3%). Monthly income also differed markedly across profiles, with Profile 1 having the highest proportion of low-income workers (0–3,000 yuan: 39.7%), whereas Profiles 3 and 4 had higher proportions in the 5,000–8,000 yuan category (26.9 and 26.8%, respectively).

**Table 3:** Demographic characteristics by latent profile [*n* (column %)].

Variables	Latent profile				Statistics
	1	2	3	4	
Age					*χ*^2^(6) = 52.273, *p* < 0.001
18–30	151 (74.0%)	691 (64.9%)	792 (54.3%)	423 (61.0%)	
31–45	36 (17.7%)	261 (24.5%)	431 (29.6%)	192 (27.7%)	
46–60	17 (8.3%)	113 (10.6%)	235 (16.1%)	78(11.3%)	
Marital status					*χ*^2^(3) = 29.977, *p* < 0.001
Unmarried	149 (73.0%)	684 (64.2%)	822 (56.4%)	420 (60.6%)	
Married	55 (27.0%)	381 (35.8%)	636 (43.6%)	273 (39.4%)	
Fertility status					*χ*^2^(3) = 41.322, *p* < 0.001
No children	165 (80.9%)	760 (71.4%)	909 (62.3%)	462 (66.7%)	
Have children	39 (19.1%)	305 (28.6%)	549 (37.7%)	231 (33.3%)	
Education					*χ*^2^(3) = 8.600, *p* = 0.035
High school and below	23 (11.3%)	140 (13.1%)	219 (15.0%)	74 (10.7%)	
College degree and above	181 (88.8%)	925 (86.9%)	1,239 (85.0%)	619 (89.3%)	
Management position					*χ*^2^(3) = 52.249, *p* < 0.001
Yes	6 (2.9%)	55 (5.2%)	180 (12.3%)	87 (9.6%)	
No	198 (97.1%)	1,010 (94.8%)	1,278 (87.8%)	606 (90.4%)	
Technical level					*χ*^2^(6) = 28.453, *p* < 0.001
Junior	133(65.2%)	679 (63.8%)	812 (55.7%)	432 (62.3%)	
Intermediate	54 (26.5%)	301 (28.3%)	456 (31.3%)	188 (27.1%)	
Advanced	17 (8.3%)	85 (8.0%)	190 (13.0%)	73 (10.5%)	
Work years					*χ*^2^(6) = 55.863, *p* < 0.001
0–10	165 (80.9%)	815 (76.5%)	945 (64.8%)	491 (70.9%)	
10–20	22 (10.8%)	141 (13.2%)	282 (19.3%)	127 (16.7%)	
>20	17 (8.3%)	109 (10.2%)	231 (15.8%)	75 (12.6%)	
Monthly income (yuan)					*χ*^2^(9) = 141.197, *p* < 0.001
0–3,000	81 (39.7%)	201 (18.9%)	192 (13.2%)	81 (11.7%)	
3,000–5,000	98 (48.0%)	630 (59.2%)	798 (54.7%)	390 (56.3%)	
5,000–8,000	19 (9.3%)	196 (18.4%)	392 (26.9%)	186 (26.8%)	
>8,000	6 (2.9%)	38 (3.6%)	76 (5.2%)	36 (5.2%)	
Average daily working hours (h)					*χ*^2^(6) = 34.727, *p* < 0.001
<8	2 (1.0%)	8 (0.8%)	25 (1.7%)	10 (1.4%)	
8	12 (5.9)	116 (10.9%)	244 (16.7%)	96 (13.9%)	
>8	190 (93.1%)	941 (88.4%)	1,189 (81.6%)	587 (84.7%)	
Number of night shifts (times)					*χ*^2^(9) = 36.639, *p* < 0.001
0	45 (22.1%)	301 (28.3%)	479 (32.9%)	255 (36.8%)	
1	5 (2.5%)	23 (2.2%)	51 (3.5%)	18 (2.6%)	
2	42 (20.6%)	254 (23.8%)	316 (21.7%)	162 (23.4%)	
≥3	112 (54.9%)	487 (45.7%)	612 (42.0%)	258 (37.2%)	

(1) Values are *n* (column %) within each latent profile. (2) Chi-square tests compare distributions across profiles.

#### Predictors of job satisfaction profile

Multinomial logistic regression (with Profile 1 as the reference) indicated that monthly income, work stress, and managerial position were the primary factors associated with profile membership. Compared with the 0–3,000 yuan group, participants earning 3,000–5,000 yuan and 5,000–8,000 yuan were significantly more likely to belong to Profiles 2, 3, and 4 (ORs > 1, *p* < 0.001). In Profile 2, the >8,000-yuan group did not reach statistical significance [OR = 2.572, 95% CI (0.984–6.723), *p* = 0.054], whereas in Profiles 3 and 4, the >8,000-yuan group remained significantly more likely to be classified into these profiles (*p* ≤ 0.01). Work stress showed profile-specific associations: it was not significantly related to membership in Profile 2 (OR = 0.998, *p* = 0.752), but was significantly negatively associated with membership in Profiles 3 and 4 [Profile 3: OR = 0.941, 95% CI (0.928–0.954); Profile 4: OR = 0.855, 95% CI (0.842–0.869); *p* < 0.001]. Holding a managerial position significantly increased the likelihood of belonging to Profiles 3 and 4 (Profile 3: OR = 3.844, *p* = 0.003; Profile 4: OR = 6.263, *p* < 0.001). In addition, compared with working >8 h per day, working <8 h per day was associated with higher odds of belonging to Profiles 2, 3, and 4 (OR = 1.875/2.937/1.995, *p* ≤ 0.049). Relative to having no night shifts, having one night shift per month was also associated with higher odds of belonging to Profiles 2, 3, and 4 (OR = 1.627/1.793/2.367, *p* ≤ 0.014), whereas higher frequencies of night shifts did not show a consistent pattern of association. Overall, demographic variables showed weak associations; only in Profile 3 was having children associated with lower odds of membership (OR = 0.487, *p* = 0.035). Additionally, in Profile 4, an intermediate technical level (vs. junior) was associated with lower odds of membership (OR = 0.514, *p* = 0.004) (see Appendix Table S5 for details).

### Demographic differences in mental health outcomes

In Table 4, we examined group differences in depression, anxiety, and digital-intelligence job insecurity across demographic characteristics.

**Table 4:** Depression, anxiety, and digital-intelligence job insecurity by demographic characteristics.

Variables	Depression			Anxiety			Digital-intelligence job insecurity		
	Mean ± SD	*t*/*F*	*p*	Mean ± SD	*t*/*F*	*p*	Mean ± SD	*t*/*F*	*p*
Age		13.244	<0.001		24.523	<0.001		4.422	0.012
18–30	6.62 ± 5.99			3.63 ± 4.67			26.09 ± 8.36		
31–45	6.89 ± 5.93			4.61 ± 4.55			26.11 ± 7.40		
46–60	7.65 ± 6.41			5.08 ± 5.05			27.17 ± 6.92		
Marital status		−4.050	<0.001		−6.248	<0.001		−1.160	0.246
Unmarried	6.19 ± 6.01			3.67 ± 4.67			26.11 ± 8.39		
Married	7.04 ± 6.08			4.70 ± 4.75			26.43 ± 7.19		
Fertility status		−2.513	0.005		−4.169	<0.001		−1.198	0.231
No children	6.32 ± 6.04			3.80 ± 4.72			26.13 ± 8.28		
Have children	6.94 ± 6.06			4.65 ± 4.68			26.46 ± 7.19		
Education		1.049	0.295		1.249	0.212		3.178	0.001
High school and below	6.82 ± 6.52			4.30 ± 5.14			27.33 ± 7.79		
College degree and above	6.48 ± 5.97			4.04 ± 4.66			26.07 ± 7.96		
Management Position		−0.850	0.396		1.629	0.103		0.127	0.899
Yes	6.27 ± 5.59			4.58 ± 4.51			26.29 ± 7.81		
No	6.55 ± 6.10			4.02 ± 4.73			26.23 ± 7.96		
Technical level		8.061	<0.001		16.974	<0.001		0.299	0.742
Junior	6.19 ± 6.07			3.71 ± 4.73			26.15 ± 8.18		
Intermediate	6.99 ± 6.01			4.53 ± 4.68			26.38 ± 7.55		
Advanced	7.14 ± 5.95			4.92 ± 4.64			26.33 ± 7.68		
Work years		11.664	<0.001		22.093	<0.001		2.950	0.053
0–10	6.21 ± 5.99			3.73 ± 4.65			26.10 ± 8.27		
10–20	7.04 ± 5.87			4.74 ± 4.56			26.25 ± 7.19		
>20	7.60 ± 6.44			5.15 ± 5.12			27.00 ± 6.91		
Monthly income (yuan)		19.035	<0.001		11.294	<0.001		0.040	0.989
0–3,000	8.24 ± 7.50			5.04 ± 5.85			26.23 ± 8.67		
3,000–5,000	6.22 ± 5.74			3.78 ± 4.48			26.26 ± 7.90		
5,000–8,000	5.99 ± 5.29			3.99 ± 4.19			26.16 ± 7.60		
>8,000	6.87 ± 6.52			4.74 ± 5.32			26.33 ± 7.61		
Average daily working hours (h)		33.098	<0.001		16.713	<0.001		2.346	0.096
<8	4.02 ± 4.43			2.84 ± 2.93			28.64 ± 8.92		
8	4.98 ± 4.78			3.19 ± 3.68			25.96 ± 8.01		
>8	6.81 ± 6.21			4.24 ± 4.67			26.24 ± 7.91		
Number of night shifts (times)		25.192	<0.001		10.897	<0.001		0.107	0.956
0	5.55 ± 5.55			3.65 ± 4.39			26.14 ± 7.94		
1	6.45 ± 6.25			4.57 ± 5.12			26.06 ± 8.43		
2	5.96 ± 5.65			3.64 ± 4.45			26.25 ± 7.96		
≥3	7.54 ± 6.43			4.59 ± 5.02			26.31 ± 7.91		
Total	6.52 ± 6.05			4.08 ± 4.73			26.24 ± 7.94		

Age was significantly associated with all three outcomes (Welch’s *F* = 13.244, 24.523, and 4.422; *p* ≤ 0.012). Marital status differed significantly in depression and anxiety (*t* = −4.050 and −6.248, *p* < 0.001) but not in digital-intelligence job insecurity (Welch’s *t* = −1.160, *p* = 0.246). Fertility status also differed significantly in depression and anxiety (*t* = −2.513, *p* = 0 0.005; *t* = −4.169, *p* < 0.001) but not in digital-intelligence job insecurity (Welch’s *t* = −1.198, *p* = 0.231). Education was significantly related to digital-intelligence job insecurity (Welch’s *t* = 3.178, *p* = 0.001) but not to depression or anxiety (*p* > 0.05), and management position was not significantly associated with any outcome (*p* > 0 0.05). In addition, technical level, work years, and monthly income showed significant group differences in depression and anxiety (*p* < 0.001), whereas none of these variables was significantly associated with digital-intelligence job insecurity (*p* > 0 0.05), including work years (*p* = 0 0.053).

### Differences in mental health outcomes across latent profiles

To examine whether mental health outcomes differed across latent profiles, we conducted one-way analyses of variance with profile membership (Profiles 1–4) as the grouping variable and depression, anxiety, and digital-intelligence job insecurity as dependent variables. Because the homogeneity of variance assumption was violated, Welch’s ANOVA was used for the omnibus tests, followed by Games–Howell *post hoc* comparisons. Results showed significant differences across the four profiles in depression, anxiety, and digital-intelligence job insecurity (*p* < 0.001). Games–Howell tests indicated a clear monotonic decrease in depression and anxiety from Profile 1 to Profile 4, with all pairwise differences significant (*p* < 0.05). For digital-intelligence job insecurity, Profile 2 showed the highest mean score and Profile 3 was intermediate, whereas Profiles 1 and 4 were lower and did not differ significantly from each other; both Profiles 1 and 4 were significantly lower than Profiles 2 and 3 (*p* < 0.05). Although the omnibus test was significant for digital-intelligence job insecurity, post-hoc comparisons showed that Profiles 1 and 4 did not differ significantly (shared superscripts), with the overall effect mainly driven by higher DI-JIS in Profiles 2 and 3 (Table 5).

**Table 5:** Depression, anxiety, and digital-intelligence job insecurity across latent profiles.

Variables	Profile 1 (*n* = 204) Mean (SD)	Profile 2 (*n* = 1,065) Mean (SD)	Profile 3 (*n* = 1,458) Mean (SD)	Profile 4 (*n* = 693) Mean (SD)	*F*	*p*
Depression	12.82 (8.62)ᵃ	9.34 (5.97)ᵇ	5.45 (4.64)ᶜ	2.59 (4.13)ᵈ	310.255	<0.001
Anxiety	8.28 (7.45)ᵃ	5.98 (4.94)ᵇ	3.31 (3.67)ᶜ	1.54 (3.14)ᵈ	206.860	<0.001
Digital-intelligence job insecurity	24.57 (10.48)ᶜ	27.59 (5.47)ᵃ	26.84 (6.83)ᵇ	23.37 (11.08)ᶜ	32.449	<0.001

Means with different superscripts (a–d) differ significantly based on Games–Howell *post hoc* tests (*p* < 0.05); means sharing the same superscript do not differ significantly.

### Hierarchical regression models predicting depression, anxiety, and digital-intelligence job insecurity

Hierarchical linear regression analyses showed that the model including only demographic and work-related characteristics explained a limited proportion of variance in depression, anxiety, and digital-intelligence job insecurity (*R*^2^ = 0.070, 0.048, and 0.005, respectively). Notably, the Step 1 increment for digital-intelligence job insecurity was not significant (Δ*F* = 1.018, *p* = 0.435). After adding work stress, the explained variance increased significantly for all three outcomes (depression Δ*R*^2^ = 0.172; anxiety Δ*R*^2^ = 0.157; digital-intelligence job insecurity Δ*R*^2^ = 0.061; *p* < 0.001). Further inclusion of the latent job satisfaction profiles produced additional incremental validity (depression Δ*R*^2^ = 0.084; anxiety Δ*R*^2^ = 0.060; digital-intelligence job insecurity Δ*R*^2^ = 0.015; *p* < 0.001). The final models explained 32.6% (adjusted *R*^2^ = 0.322) of the variance in depression, 26.5% (adjusted *R*^2^ = 0.260) in anxiety, and 8.1% (adjusted *R*^2^ = 0.075) in digital-intelligence job insecurity (see Appendix Table S6).

In the final models, work stress was positively associated with depression [*B* = 0.127, 95% CI (0.112, 0.143)], anxiety [*B* = 0.105, 95% CI (0.092, 0.117)], and digital-intelligence job insecurity [*B* = 0.138, 95% CI (0.114, 0.161); *p* < 0.001]. With Profile 1 as the reference, Profiles 2–4 reported significantly lower depression and anxiety (depression: *B* = −3.031/−5.979/−7.261; anxiety: *B* = −2.109/−4.143/−4.629; *p* < 0.001). In contrast, digital-intelligence job insecurity was significantly higher in Profiles 2 and 3 (*B* = 3.086 and 3.287; *p* < 0.001), and also higher in Profile 4, although the effect size was smaller [*B* = 1.361, 95% CI (0.061, 2.661), *p* = 0.040]. Beyond the focal predictors, several covariates remained significant; the most consistent were monthly income and night-shift frequency for depression/anxiety, and education and daily working hours for digital-intelligence job insecurity (Table 6).

**Table 6:** Hierarchical linear regression results for mental health outcomes.

Variables	Variables	Depression		Anxiety		Digital-intelligence job insecurity	
		*B* (95%CI)	*p*	*B* (95%CI)	*p*	*B* (95%CI)	*p*
Intercept		4.06 (1.47, 6.66)	0.002	2.94 (0.82, 5.05)	0.007	23.03 (19.06, 27.01)	<0.001
Age	31–45	0.82 (0.18, 1.45)	0.012	0.66 (0.14, 1.18)	0.013	−0.19 (−1.16, 0.79)	0.708
	46–60	1.3 (−0.01, 2.62)	0.052	0.45 (−0.62, 1.52)	0.412	0.68 (−1.34, 2.69)	0.511
Marital status	Married	0.44 (−0.28, 1.16)	0.229	0.41 (−0.17, 1)	0.166	−0.13 (−1.23, 0.97)	0.816
Fertility	Have children	−0.64 (−1.35, 0.07)	0.078	−0.4 (−0.98, 0.18)	0.18	0.09 (−1.01, 1.18)	0.876
Education status	College degree and above	0.34 (−0.21, 0.89)	0.228	0.21 (−0.24, 0.65)	0.368	−0.93 (−1.77, −0.09)	0.029
Management position	No	0.48 (−0.15, 1.11)	0.132	−0.09 (−0.61, 0.42)	0.722	0.08 (−0.88, 1.05)	0.864
Technical level	Intermediate	0.39 (−0.05, 0.82)	0.086	0.27 (−0.09, 0.63)	0.135	0.08 (−0.6, 0.75)	0.823
	Advanced	0.63 (0, 1.25)	0.049	0.64 (0.13, 1.15)	0.014	−0.03 (−0.99, 0.93)	0.956
Work years	10–20	0.86 (0.24, 1.49)	0.007	0.66 (0.15, 1.17)	0.011	0.18 (−0.78, 1.14)	0.713
	>20	0.73 (−0.51, 1.98)	0.247	1.01 (−0.01, 2.02)	0.052	−0.51 (−2.41, 1.4)	0.6
Monthly income	3,000–5,000	−1.09 (−1.57, −0.6)	<0.001	−0.57 (−0.96, −0.18)	0.005	0.34 (−0.4, 1.08)	0.37
	5,000–8,000	−1.53 (−2.11, −0.94)	<0.001	−0.67 (−1.15, −0.19)	0.007	0.24 (−0.66, 1.14)	0.6
	>8,000	−1.24 (−2.18, −0.29)	0.01	−0.5 (−1.27, 0.27)	0.204	0.27 (−1.18, 1.72)	0.713
Average daily working hours	8 h	0.92 (−0.61, 2.45)	0.239	0.24 (−1.01, 1.49)	0.701	−2.64 (−4.99, −0.29)	0.028
	<8 h	1.63 (0.15, 3.11)	0.031	0.52 (−0.69, 1.73)	0.4	−2.54 (−4.8, −0.27)	0.028
Number of night shifts	1	0.81 (−0.23, 1.85)	0.125	0.93 (0.09, 1.78)	0.031	−0.31 (−1.9, 1.28)	0.703
	2	0.42 (−0.05, 0.88)	0.08	0.05 (−0.33, 0.43)	0.794	0.15 (−0.57, 0.86)	0.689
	≥3	1.31 (0.91, 1.71)	<0.001	0.5 (0.17, 0.83)	0.003	−0.09 (−0.71, 0.52)	0.769
Work stress	Work stress	0.13 (0.11, 0.14)	<0.001	0.11 (0.09, 0.12)	<0.001	0.14 (0.11, 0.16)	<0.001
Job satisfaction	Profile 2	−3.03 (−3.79, −2.28)	<0.001	−2.11 (−2.73, −1.49)	<0.001	3.09 (1.93, 4.24)	<0.001
	Profile 3	−5.98 (−6.74, −5.22)	<0.001	−4.14 (−4.76, −3.52)	<0.001	3.29 (2.12, 4.45)	<0.001
	Profile 4	−7.26 (−8.11, −6.41)	<0.001	−4.63 (−5.32, −3.94)	<0.001	1.36 (0.06, 2.66)	0.04

(1) Coefficients are from the final (Step 3) model. (2) Job satisfaction profile membership is entered as dummy variables with Profile 1 as the reference. (3) Reference groups: Age = 18–30; Marital status = Unmarried; Fertility = No children; Education = High school and below; Management position = Yes; Technical level = Junior; Work years = 0–10; Monthly income = 0–3,000; Daily working hours= > 8 h; Night shifts = 0.

## Discussion

This study used a person-centered approach to identify latent profiles of job satisfaction and examined their associations with depressive symptoms, anxiety symptoms, and digital-intelligence job insecurity. Three take-home messages emerged. First, job satisfaction was heterogeneous and could be summarized into four level-graded profiles. Second, depressive and anxiety symptoms showed a clear gradient across profiles, decreasing as job satisfaction increased. Third, digital-intelligence job insecurity displayed a non-monotonic pattern across profiles, suggesting that technology-related threat appraisals do not map linearly onto overall job satisfaction. Across outcomes, work stress was consistently associated with symptom levels and contributed the largest incremental explanatory power.

The overall mean job satisfaction score (3.62 ± 0.90) aligns with prior research findings (58, 59) suggesting that the job satisfaction of industrial workers remains moderate across diverse industrial contexts. Four job satisfaction profiles were broadly similar in response patterns across items, and the primary differences reflected a clear gradient in overall level (very low, low-to-moderate, moderately high, high), but the item-level patterns revealed several representative differences. First, salary-related satisfaction (Q13) was relatively low across all four profiles and was especially salient among the lower-satisfaction groups. This pattern is consistent with prior evidence highlighting the importance of pay for job satisfaction and suggests that pay may represent a relatively stable area of lower satisfaction in frontline manufacturing jobs (60). For example, studies have found significant linear relationships between salary income and job satisfaction (61), with salary and rewards identified as key factors in the construction industry (62). Second, job stability (Q8) showed comparatively higher item-level scores in the low-satisfaction groups. This suggests that when overall satisfaction is low, workers’ evaluation of “stable employment” may function as a key compensatory resource or psychological anchor: even if they are dissatisfied with rewards and development opportunities, perceived stability may still support their baseline appraisal of the job. This finding aligns with prior work suggesting that job stability contributes uniquely to job satisfaction and becomes more salient under uncertain conditions (63). This finding aligns with broader research indicating that stable employment directly enhances job satisfaction, whereas job instability can negatively affect employees’ overall workplace contentment (64, 65). In addition, as the profile level increased (Profiles 3 and 4), item scores rose overall and became more balanced; items related to professional ethics and perceived competence (e.g., Q7 and Q11) remained relatively high in the moderately high and high satisfaction groups, consistent with evidence that work ethics and positive work attitudes are associated with higher job satisfaction (66).

In the analyses of profile differences and predictors, we found that membership in job satisfaction profiles was stably associated with several structural factors. Specifically, income was the most consistent differentiating factor: higher-income workers were significantly more likely to belong to Profiles 2–4, which accords with the well-established association between pay and job satisfaction (67, 68). Within the JD–R framework, income can be viewed as an important job resource or a form of resource compensation; higher income typically implies stronger returns and greater resource replenishment, which is consistent with higher job satisfaction patterns (69, 70). Second, holding a managerial position was associated with a higher likelihood of membership in higher job satisfaction profiles, which may reflect advantages in autonomy, decision latitude, and access to resources (71, 72). Work stress was negatively associated with membership in the higher-satisfaction profiles, particularly Profiles 3–4, whereas its association with Profile 2 was not significant. This is consistent with the JD–R proposition that high demands deplete resources, elicit strain responses, and undermine positive work experiences (73), and has been supported by prior empirical evidence (74–76). Shorter daily working hours were associated with greater odds of belonging to Profiles 2–4, consistent in direction with evidence that long working hours can constrain recovery and reduce positive work experiences (77, 78). Regarding night shifts, a positive association was observed only for “one night shift per month,” whereas higher frequencies did not show a linear gradient. This pattern may reflect the coexistence of compensatory resources (e.g., shift premiums or scheduling arrangements) and health-related depletion in shift work (79, 80), resulting in a non-linear association with job satisfaction profile membership.

Several demographic and job-related characteristics showed independent associations with the mental health indicators. For depression and anxiety, higher income was associated with lower levels of depression and anxiety, whereas the adverse association between night shifts and mental health was mainly observed in the context of high-frequency night shifts. We consider several plausible explanations. First, greater workload and insufficient recovery: night shifts and disrupted circadian rhythms may reduce sleep and recovery time, increasing the risk of emotional distress (81–84). Second, differences in job roles and work arrangements: night shifts are often concentrated in specific tasks or positions, where work intensity, exposure risks, and supervisory requirements may differ from regular day shifts (84–86). An individual’s health status, family responsibilities, or prior adaptability may influence whether they are assigned to night/rotating shifts, making the direction of associations more complex (87, 88). Because this study is cross-sectional, these explanations should be tested further in longitudinal or quasi-experimental research.

In this study, daily working hours were negatively associated with digital-intelligence job insecurity, suggesting that longer working hours do not necessarily correspond to higher perceived digital-intelligence threat. One possible interpretation is that, in this organizational context, longer working hours may be more common in positions that rely more heavily on manual operations. In contrast, positions with shorter working hours may involve more frequent collaboration with automated equipment, making concerns about job replacement, task transformation, and changing requirements for human–machine collaboration more salient (89, 90). From an Industry 5.0 perspective, this pattern is consistent with a human–machine collaboration shift in which technology exposure is uneven across job roles; digital-intelligence job insecurity may therefore concentrate in positions where smart systems more directly redesign daily tasks rather than in roles characterized primarily by long manual hours. At the same time, the negative association between education level and digital-intelligence job insecurity is also consistent with an “adaptive resources” pathway. Higher education may be linked to better technical understanding, learning and working capacity, or self-efficacy (91–93), which could reduce the perceived threat posed by digital-intelligence-related changes. This interpretation aligns with Industry 5.0’s emphasis on continuous reskilling/upskilling and higher skill requirements for working alongside intelligent systems: workers with stronger learning resources and perceived employability may feel better prepared for digitally redesigned tasks and thus report lower digital-intelligence job insecurity.

Consistent with prior studies, our investigation revealed significant mental health concerns among manufacturing workers, with mean scores of 6.52 ± 6.05 for depression, 4.08 ± 4.73 for anxiety, and 26.24 ± 7.94 for digital-intelligence job insecurity. These scores align with findings from male workers in small manufacturing settings (depression: 6.37) (18) and Pakistani medical industry workers (depression: 6.89 ± 6.64) (94). Similar anxiety ratings have been observed in other populations, such as Korean nurses (anxiety: 4.0) (95).

While prior research has shown that artificial intelligence can increase job insecurity through identity threats (96), our study underscores this issue’s nuanced relationship with other mental health outcomes: depression and anxiety followed a clear gradient across profiles, with the very-low satisfaction group showing the greatest symptom burden, whereas digital-intelligence job insecurity was highest in the low-to-moderate profile and remained relatively high in the moderately high profile, indicating that digital-intelligence–related threat appraisals may not map linearly onto overall job satisfaction.

After controlling for demographic and job-related characteristics and further adjusting for work stress, the hierarchical linear regression results showed that job satisfaction profiles showed a stable “level gradient” association with depression and anxiety. This indicates that moving from very low satisfaction to high satisfaction is consistently associated with a lower levels of depression and anxiety (40, 97). Work stress was significantly and positively associated with all three outcomes (98), suggesting that stress-related experiences may be an important proximal correlate of psychological distress among frontline manufacturing workers and may play a key role in the pathway linking work conditions to mental health (99, 100).

Unlike depression and anxiety, digital-intelligence job insecurity showed a non-linear association with job satisfaction profiles, peaking in the mid-satisfaction profiles, suggesting that DI-JIS is not simply a function of global dissatisfaction but is also shaped by technology-transition exposure and perceived skill–task mismatch. This pattern aligns with the Industry 5.0 emphasis on human-centric digital transformation, where human–machine collaboration and continuous reskilling become central to everyday work. One possible explanation is that digital-intelligence job insecurity captures concerns related to digital-intelligence changes, including job replacement, task transformation, and human–machine collaboration, which often require some degree of technological exposure and concrete, observable change (101). Workers in the moderate-satisfaction profiles may remain actively involved in day-to-day operations and thus have more frequent exposure to digital systems. In an Industry 5.0 transition, these workers may be in a “high-exposure, high-uncertainty” zone: they experience frequent workflow redesign and new digital demands, yet may not fully perceive sufficient training access, role clarity, or internal mobility signals to feel secure. Technostress research suggests that repeated system upgrades and workflow changes can create techno-uncertainty, making digital change feel less predictable and more salient (102). If these workers have not yet developed stable routines or sufficient adaptive resources to buffer ongoing changes, digital-intelligence job insecurity may be more pronounced in the moderate-satisfaction profiles. From the perspective of Herzberg’s two-factor theory, workers in the very low job satisfaction profile may be primarily preoccupied with hygiene factors such as pay, working conditions, and organizational policies. When these hygiene factors are perceived as insufficient, they tend to generate strong dissatisfaction and become the most salient focus of evaluation (103). As a result, concerns related to digital-intelligence–driven change may be comparatively less prioritized in these workers’ subjective appraisal, because their attention is anchored to more immediate and pressing shortcomings in the work environment.

Meanwhile, profile 4 showed the smallest increase in digital-intelligence job insecurity relative to the reference profile, indicating the weakest association among the non-reference profiles. This pattern is consistent with the resource-buffering perspective of Conservation of Resources (COR) theory (104). Workers with higher satisfaction may have greater psychological and organizational resources, which can attenuate the perceived threat associated with digital transformation (105). Importantly, an Industry 5.0 lens suggests that such resources often include access to reskilling opportunities, supportive leadership during human–machine collaboration, and clearer signals of career continuity in digitally redesigned roles, all of which are factors that can reduce digital-intelligence job insecurity even under substantial technology exposure. At the same time, the persistence of a small effect suggests that high satisfaction does not eliminate technology-related concerns; rather, it may reflect a more realistic awareness of changing job requirements while remaining relatively well-resourced.

Overall, H1 was supported: latent profile analysis identified four distinct job satisfaction profiles. H2 was supported for all three outcomes: depression and anxiety showed a clear gradient across profiles, whereas DI-JIS differed across profiles in a non-monotonic pattern.

### Theoretical and practical implications

#### Theoretical implications

This study extends the job satisfaction–mental health literature by demonstrating that job satisfaction is not a homogeneous construct in frontline manufacturing workers undergoing Industry 5.0 transformation. A person-centered perspective reveals meaningful heterogeneity that would be masked by variable-centered “average” associations: depressive and anxiety symptoms showed a clear gradient across four job satisfaction profiles, whereas digital-intelligence job insecurity followed a non-monotonic pattern (peaking in mid-satisfaction profiles rather than the very-low profile). This configuration suggests that technology-related insecurity is partly distinct from general affective evaluations of work and should not be assumed to change linearly with overall job satisfaction. Together with the substantial contribution of work stress, these findings support a differentiated view in which general strain processes and technology-transition concerns can co-exist but do not fully overlap.

#### Practical implications

The profile-specific patterns provide actionable guidance for targeted interventions. Workers in the very-low satisfaction profile showed the greatest burden of depressive and anxiety symptoms and may therefore benefit most from priority mental health screening and support, alongside workload and recovery management (e.g., reducing excessive work hours, stabilizing schedules, and improving rest opportunities). In contrast, the elevated digital-intelligence job insecurity observed in the mid-satisfaction profiles suggests a need for technology-transition support. Clear communication about technological change, transparent reskilling pathways, and accessible training opportunities may be particularly relevant for these workers. Overall, combining stress-reduction strategies with profile-informed, targeted support may improve the precision and efficiency of workplace health promotion during digital transformation.

## Conclusion

This study examined heterogeneity in job satisfaction among frontline industrial workers and its associations with mental health outcomes. Latent profile analysis identified four job satisfaction profiles. Job satisfaction profile membership remained strongly associated with depression and anxiety. Digital-intelligence job insecurity showed a non-monotonic pattern across profiles. These findings suggest that an individual-centered profile approach provides actionable differentiation of mental health symptom burden across distinct job satisfaction patterns, supporting more targeted workplace strategies.

### Limitations and future research

Despite the rigorous design of this study, here are several limitations. The cross-sectional design employed in this study only revealed the association between job satisfaction and depression, anxiety, and digital-intelligence job insecurity; however, it fails to determine causal direction, specifically regarding whether low job satisfaction leads to depression or if the opposite is true. Future research should adopt longitudinal designs, intervention or quasi-experimental designs in organizational settings to better identify temporal ordering and causal mechanisms. Second, the sample of this study consists solely of male employees from a large automobile manufacturing enterprise in Jilin Province, China, and does not include female workers. This makes it difficult to generalize the research findings to other manufacturing industries (such as electronics manufacturing, mechanical processing, etc.), private enterprises, or small and medium-sized industrial enterprises, it may still limit the generalizability of the research results. Future studies could expand the sample source to include a broader population (such as multi-industry, multi-regional, and female workers).

## Data Availability

The raw data supporting the conclusions of this article will be made available by the authors, without undue reservation.
