# Supplementary material for: Profiles of job satisfaction among industrial workers and its association with mental health under the background of Industry 5.0 transformation: a latent profile analysis
Source: Front Public Health. 2026 Mar 6;14:1772767. doi: 10.3389/fpubh.2026.1772767 (PMC13002619; doi:10.3389/fpubh.2026.1772767)
Supplement: Supplementary file 1 [file Data_Sheet_1.docx]

**Appendix**

**Appendix Table S1：Results of Confirmatory Factor Analysis and Convergent Validity**

**Table S1 Confirmatory Factor Analysis and Convergent Validity**

| **Constructs / Items** | **Standardized Loading** | **CR** | **AVE** |
| --- | --- | --- | --- |
| **Digital Intelligence Job Insecurity** |  | 0.930 | 0.816 |
| **Job Replacement Insecurity** | 0.850^a***^ | 0.889 | 0.731 |
| **Item 1** | 0.689^***^ |  |  |
| **Item 2** | 0.905^***^ |  |  |
| **Item 3** | 0.948^***^ |  |  |
| **Job Transformation Insecurity** | 0.920^a***^ | 0.943 | 0.846 |
| **Item 4** | 0.920^***^ |  |  |
| **Item 5** | 0.917^***^ |  |  |
| **Item 6** | 0.922^***^ |  |  |
| **Job Interaction Insecurity** | 0.938^a***^ | 0.862 | 0.675 |
| **Item 7** | 0.833^***^ |  |  |
| **Item 8** | 0.852^***^ |  |  |
| **Item 9** | 0.778^***^ |  |  |

*Note: *** p <0.001. CR = Composite Reliability; AVE = Average Variance Extracted.^a^ Represents the second-order factor loadings.*

**Appendix Table S2：Results of Discriminant Validity Analysis**

**Table S2：Discriminant Validity: Fornell-Larcker Criterion and HTMT Ratios**

| **Constructs** | **1** | **2** | **3** |
| --- | --- | --- | --- |
| **Job Replacement Insecurity** | **0.855** | 0.814 | 0.807 |
| **Job Transformation Insecurity** | 0.732 | **0.920** | 0.846 |
| **Job Interaction Insecurity** | 0.705 | 0.768 | **0.822** |

*Note. Diagonal elements (bold) are the square roots of AVE. Lower-triangular elements are inter-construct correlations. Upper-triangular elements are HTMT ratios. Discriminant validity is supported when √AVE exceeds inter-construct correlations and HTMT <0 .85.*

**Appendix Table S3：Result of Model Fit Indices of Digital-Intelligence Job Insecurity**

**Table S3 Model Fit Indices of Digital-Intelligence Job Insecurity**

| **Model** | **χ^2^** | **df** | **χ^2^/df** | **CFI** | **TLI** | **RMSEA** | **SRMR** |
| --- | --- | --- | --- | --- | --- | --- | --- |
| **Second-Order Model** | 344.349 | 21 | 16.398 | 0.988 | 0.980 | 0.067 | 0.018 |

**Appendix Table S4：Class counts and proportions for the 2–5 profile solutions**

**Table S4 Class counts and proportions for the 2–5 profile solutions**

| **K=2** | **n** | **%** |
| --- | --- | --- |
| Class 1 | 1388 | 40.58 |
| Class 2 | 2032 | 59.42 |
| **K=3** |  |  |
| Class 1 | 1124 | 32.87 |
| Class 2 | 1601 | 46.81 |
| Class 3 | 695 | 20.32 |
| **K=4** |  |  |
| Class 1 | 204 | 5.97 |
| Class 2 | 1065 | 31.14 |
| Class 3 | 1458 | 42.63 |
| Class 4 | 693 | 20.26 |
| **K=5** |  |  |
| Class 1 | 108 | 3.15 |
| Class 2 | 204 | 5.97 |
| Class 3 | 1011 | 29.56 |
| Class 4 | 689 | 20.15 |
| Class 5 | 1408 | 41.17 |

**Appendix Table S5. Multinomial logistic regression predicting job satisfaction profile**

**Table S5 Multinomial logistic regression predicting job satisfaction profile**

| Latent Profile | Variables | Control  group | Variables | B | *P* | OR | 95%CI | |
| --- | --- | --- | --- | --- | --- | --- | --- | --- |
|  |  |  |  |  |  |  | Lower limit | Upper limit |
| 2 | Work stress |  |  | -0.002 | 0.752 | 0.998 | 0.985 | 1.011 |
|  |  | 18-30 |  |  |  |  |  |  |
|  |  |  | 31-45 | 0.204 | 0.489 | 1.226 | 0.688 | 2.187 |
|  |  |  | 46-60 | 0.388 | 0.56 | 1.475 | 0.399 | 5.444 |
|  | Marital Status |  | Unmarried | 0.065 | 0.831 | 1.067 | 0.588 | 1.937 |
|  | Fertility Status |  | No children | -0.584 | 0.086 | 0.557 | 0.286 | 1.085 |
|  | Education |  | High school  and below | 0.178 | 0.504 | 1.194 | 0.71 | 2.01 |
|  | Management  Position |  | Yes | 0.377 | 0.413 | 1.458 | 0.591 | 3.598 |
|  | Technical level | Junior |  |  |  |  |  |  |
|  |  |  | Intermediate | -0.325 | 0.114 | 0.722 | 0.483 | 1.081 |
|  |  |  | Advanced | -0.404 | 0.194 | 0.668 | 0.363 | 1.228 |
|  | Work years | 0-10 |  |  |  |  |  |  |
|  |  |  | 10-20 | -0.341 | 0.302 | 0.711 | 0.372 | 1.359 |
|  |  |  | >20 | -0.59 | 0.363 | 0.554 | 0.155 | 1.978 |
|  | Monthly income  (yuan) | 0-3000 |  |  |  |  |  |  |
|  |  |  | 3000-5000 | 0.987 | 0 | 2.683 | 1.904 | 3.781 |
|  |  |  | 5000-8000 | 1.451 | 0 | 4.265 | 2.389 | 7.616 |
|  |  |  | >8000 | 0.945 | 0.054 | 2.572 | 0.984 | 6.723 |
|  | Average daily  working hours | >8h | 8h | -0.05 | 0.95 | 0.951 | 0.196 | 4.622 |
|  |  |  | <8h | 0.628 | 0.049 | 1.875 | 1.001 | 3.509 |
|  | Number of  night shifts | 0 | 1 | 0.487 | 0.014 | 1.627 | 1.104 | 2.399 |
|  |  |  | 2 | 0.249 | 0.627 | 1.283 | 0.469 | 3.506 |
|  |  |  | ≥3 | 0.313 | 0.119 | 1.368 | 0.923 | 2.027 |
| 3 | Work stress |  |  | -0.061 | 0 | 0.941 | 0.928 | 0.954 |
|  | Age | 18-30 |  |  |  |  |  |  |
|  |  |  | 31-45 | 0.286 | 0.338 | 1.331 | 0.742 | 2.387 |
|  |  |  | 46-60 | 0.76 | 0.253 | 2.137 | 0.581 | 7.859 |
|  | Marital Status |  | Unmarried | 0.401 | 0.197 | 1.493 | 0.812 | 2.744 |
|  | Fertility Status |  | No children | -0.72 | 0.035 | 0.487 | 0.249 | 0.951 |
|  | Education |  | High school  and below | 0.251 | 0.344 | 1.286 | 0.764 | 2.163 |
|  | Management  Position |  | Yes | 1.346 | 0.003 | 3.844 | 1.593 | 9.276 |
|  | Technical level | Junior |  |  |  |  |  |  |
|  |  |  | Intermediate | -0.366 | 0.078 | 0.694 | 0.462 | 1.042 |
|  |  |  | Advanced | -0.14 | 0.649 | 0.87 | 0.477 | 1.586 |
|  | Work years | 0-10 |  |  |  |  |  |  |
|  |  |  | 10-20 | 0.003 | 0.992 | 1.003 | 0.526 | 1.915 |
|  |  |  | >20 | -0.267 | 0.679 | 0.766 | 0.216 | 2.715 |
|  | Monthly income  (yuan) | 0-3000 |  |  |  |  |  |  |
|  |  |  | 3000-5000 | 1.201 | 0 | 3.322 | 2.333 | 4.731 |
|  |  |  | 5000-8000 | 2.008 | 0 | 7.447 | 4.17 | 13.297 |
|  |  |  | >8000 | 1.238 | 0.01 | 3.449 | 1.336 | 8.9 |
|  | Average daily  working hours | >8h | 8h | 0.933 | 0.219 | 2.543 | 0.574 | 11.279 |
|  |  |  | <8h | 1.077 | 0.001 | 2.937 | 1.58 | 5.459 |
|  | Number of  night shifts | 0 | 1 | 0.584 | 0.003 | 1.793 | 1.215 | 2.645 |
|  |  |  | 2 | 0.789 | 0.114 | 2.202 | 0.827 | 5.861 |
|  |  |  | ≥3 | 0.2 | 0.327 | 1.221 | 0.819 | 1.82 |
| 4 | Work stress |  |  | -0.156 | 0 | 0.855 | 0.842 | 0.869 |
|  | Age | 18-30 |  |  |  |  |  |  |
|  |  |  | 31-45 | 0.151 | 0.654 | 1.163 | 0.601 | 2.252 |
|  |  |  | 46-60 | 0.787 | 0.294 | 2.197 | 0.504 | 9.567 |
|  | Marital Status |  | Unmarried | 0.2 | 0.574 | 1.221 | 0.609 | 2.449 |
|  | Fertility Status |  | No children | -0.668 | 0.079 | 0.513 | 0.243 | 1.08 |
|  | Education |  | High school  and below | -0.083 | 0.784 | 0.921 | 0.509 | 1.665 |
|  | Management  Position |  | Yes | 1.835 | 0 | 6.263 | 2.492 | 15.737 |
|  | Technical level | Junior |  |  |  |  |  |  |
|  |  |  | Intermediate | -0.665 | 0.004 | 0.514 | 0.325 | 0.812 |
|  |  |  | Advanced | -0.41 | 0.228 | 0.664 | 0.341 | 1.291 |
|  | Work years | 0-10 |  |  |  |  |  |  |
|  |  |  | 10-20 | -0.036 | 0.92 | 0.965 | 0.476 | 1.953 |
|  |  |  | >20 | -0.603 | 0.405 | 0.547 | 0.133 | 2.26 |
|  | Monthly income  (yuan) | 0-3000 |  |  |  |  |  |  |
|  |  |  | 3000-5000 | 1.334 | 0 | 3.796 | 2.465 | 5.848 |
|  |  |  | 5000-8000 | 2.269 | 0 | 9.673 | 5.071 | 18.451 |
|  |  |  | >8000 | 1.586 | 0.003 | 4.883 | 1.729 | 13.791 |
|  | Average daily  working hours | >8h | 8h | 0.769 | 0.354 | 2.158 | 0.424 | 10.99 |
|  |  |  | <8h | 0.691 | 0.041 | 1.995 | 1.03 | 3.865 |
|  | Number of  night shifts | 0 | 1 | 0.862 | 0 | 2.367 | 1.542 | 3.634 |
|  |  |  | 2 | 0.683 | 0.226 | 1.98 | 0.655 | 5.985 |
|  |  |  | ≥3 | 0.235 | 0.303 | 1.265 | 0.809 | 1.978 |

*Note. Profile 1 was the reference category.*

**Appendix Table S6. Hierarchical regression model summary**

Table S6 Hierarchical regression model summary

| **Outcome** | Step 1 | Step 2 | Step 3 | Final R² | Final Adjusted R² |
| --- | --- | --- | --- | --- | --- |
|  | R² | ΔR² | ΔR² |  |  |
| Depression | 0.070 | 0.172*** | 0.084*** | 0.326 | 0.322 |
| Anxiety | 0.048 | 0.157*** | 0.060*** | 0.265 | 0.260 |
| Digital-intelligence job insecurity | 0.005 | 0.061*** | 0.015*** | 0.081 | 0.075 |

*Notes: Step 1 includes demographic and work-related covariates. Step 2 adds work stress. Step 3 adds job satisfaction profile membership (Profile 1 as reference)*
